# Prevalence and Antibiotic Susceptibility of Community-Associated Methicillin-Resistant *Staphylococcus aureus* in a Rural Area of India: Is MRSA Replacing Methicillin-Susceptible *Staphylococcus aureus* in the Community?

**DOI:** 10.5402/2012/248951

**Published:** 2012-10-15

**Authors:** Gerardo Alvarez-Uria, Raghuprakash Reddy

**Affiliations:** ^1^Department of Infectious Diseases, Rural Development Trust Hospital, Kadiri Road, Bathalapalli 515661, India; ^2^Department of Microbiology, Rural Development Trust Hospital, Kadiri Road, Bathalapalli 515661, India

## Abstract

*Staphylococcus aureus* (SA) is the most common cause of skin and soft tissue infections (SSTIs) and nosocomial infections. In developed countries there is a major concern about the rise of community-associated methicillin-resistant SA (CA-MRSA), but data from developing countries are scarce. In this study we describe the prevalence and antibiotic susceptibility of CA-MRSA and healthcare-associated MRSA (HA-MRSA) in a district hospital from rural India. We identified 119 CA-SA infections and 82 HA-SA infections. The majority of infections were SSTI, and the proportion of MRSA in CA-SA and HA-SA infections was 64.7% and 70.7%, respectively. The proportion of CA-MRSA in children <5 years was 73.7%. We did not observe any linezolid or vancomycin resistance. CA-SA had high levels of resistance to ciprofloxacin and low levels of resistance to chloramphenicol, doxycycline, rifampicin, and clindamycin. CA-MRSA had higher proportion of resistance to ciprofloxacin, erythromycin, gentamicin, and cotrimoxazole than CA methicillin-susceptible SA (CA-MSSA). HA-MRSA had higher proportion of resistance to clindamycin and doxycycline than CA-MRSA. The results of this study indicate that MRSA is replacing MSSA in CA-SA infections. If these findings are confirmed by other studies, the spread of CA-MRSA can be a major public health problem in India.

## 1. Introduction

Although *Staphylococcus aureus* (SA) is a commensal organism, persistently or transiently present in up to 80% of healthy individuals, it is the most common cause of skin and soft tissue infections (SSTIs) and nosocomial infections, and it is able to produce necrotizing pneumonia, septic arthritis, endocarditis, and osteomyelitis [1, 2]. Before the antibiotic era, the mortality of blood stream infections caused by SA was above 80% [3]. After the discovery of penicillin, the mortality of SA infections was reduced dramatically [4]. However, soon after the introduction of penicillin, penicillinase-producer SA strains were described. These penicillin-resistant strains spread into hospitals and years later into the community [4]. Penicillin-resistant strains became more common than penicillin-susceptible strains, first in hospitals and later in the community, by the late 1970s [2]. Antibiotic drugs that were effective against penicillinase-producer strains such as methicillin became the drugs of choice for treating SSTI and other SA-related infections. However, soon after the introduction of methicillin, SA strains with a modified transpeptidase that had low affinity for beta-lactam antibiotics were described [2]. These methicillin-resistant SA (MRSA) strains spread during the 1990s into healthcare facilities in the United Kingdom and other parts of the world [2, 5]. Nowadays, the prevalence of health-care associated MRSA (HA-MRSA) is higher than health-care associated methicillin-susceptible SA (HA-MSSA) in some countries of America and Asia [6]. Although the prevalence of community associated MRSA (CA-MRSA) remains low in some European countries, there is increasing evidence that CA-MRSA is on the rise in many parts of the world [7].

The burden of common bacterial infections is higher in low- and middle-income countries [8], but data about the prevalence of MRSA in these countries are scarce [7, 9, 10]. While MRSA infections are associated with increased mortality and costs for healthcare systems in developed countries [2], the spread of MRSA in resource-limited settings can have devastating consequences because of the lack of microbiology laboratories that can provide bacterial identification and antimicrobial susceptibilities and the high cost of antibiotic drugs required to treat severe MRSA infections [8]. 

MRSA is mainly transmitted through skin-to-skin contact [2]. India is a country with high population density, where the antibiotic consumption in humans is extremely high and there is no regulation of the use of antibiotics in livestock and poultry [11]. This combination makes a perfect scenario for the spread of drug-resistant bacteria in the community [11, 12]. Although the prevalence of HA-MRSA has been described in some Indian hospitals [11], the prevalence of CA-MRSA in India is not well known. The aim of this study is to describe the prevalence and antibiotic susceptibility patterns of CA-MRSA in a district hospital situated in a rural area of India.

## 2. Methods

The study was performed in the Bathalapalli Rural Development Trust (RDT) Hospital, a nonprofit private district hospital situated in a rural area of Andhra Pradesh, India. The hospital provides free consultation and medicines at reduced cost to people of low socioeconomic status. 

For this study we included all SA-infections diagnosed in the hospital from 24th March 2011 to 17th August 2012. Antimicrobial susceptibilities were performed using disk diffusion testing according to recommendations of the Clinical Laboratory Standard Institute [13]. Inducible clindamycin resistance was tested by double disk diffusion (*D* test) [14]. Methicillin resistance was tested by cefoxitin disk diffusion [15]. CA-SA was defined according to Centers for Disease Control and Prevention (CDC) definition for CA-MRSA when patients did not meet any of the following criteria: (i) isolation of SA more than 48 hours after hospital admission; (ii) history of hospitalization, surgery, dialysis, or residence in a long-term care facility within one year of the SA culture; (iii) presence of an indwelling catheter or a percutaneous device at the time of culture; (iv) previous isolation of MRSA [16]. 

Statistical analysis was performed using Stata Statistical Software (Stata Corporation. Release 11. College Station, TX, USA). Chi-square test was used for comparing proportions. We calculated confidence intervals for proportions to allow for sampling error. The study was approved by the RDT Hospital Ethical Committee.

## 3. Results

We identified 119 community-associated and 82 health-care-associated infections produced by SA. The median age of patients was 29 years (interquartile range, 8.5 to 39.3), and 60.2% were males. CA-SA was isolated from pus or exudates from SSTI (70%), blood culture (12%), sputum (9%), urine (2%), and other specimens (7%). HA-SA was isolated from pus or exudates from SSTI (67%), blood culture (23%), sputum (5%), urine (2%), and other specimens (3%). The proportion of MRSA in CA-SA infections was 64.7% (95% confidence interval (CI), 55.8 to 72.7), and the proportion of MRSA in HA-SA infections was 70.7% (95% CI, 60.1 to 79.4). The proportion of HA-MRSA was not significantly higher than the proportion of CA-MRSA (*P* = 0.37). The proportion of children <5 years with MRSA in CA-SA infections was 73.7% (95% CI, 51.2 to 88.2). We did not find any vancomycin-resistant strain, but 6/196 (3.1%, 95% CI, 1.4 to 6.5) were intermediate resistant to vancomycin. Linezolid susceptibility was tested for 33 MRSA strains, and all of them were linezolid susceptible. 

Comparison of antibiotic susceptibilities to ciprofloxacin, erythromycin, clindamycin, doxycycline, gentamicin, rifampicin, trimethoprim/sulfamethoxazole (cotrimoxazole), chloramphenicol, and vancomycin is presented in Table 1. We found high levels of resistance to ciprofloxacin in CA-SA and HA-SA infections. Most of CA-SA strains were susceptible to chloramphenicol, doxycycline, rifampicin, clindamycin, and gentamicin. HA-SA had higher proportion of resistance to clindamycin, and doxycycline than CA-SA. When comparing CA-MSSA and HA-MSSA, we did not find any significant difference in antibiotic susceptibilities. HA-MRSA had higher proportion of resistance to clindamycin and doxycycline than CA-MRSA. Globally, MRSA had higher proportion of resistance to all antibiotics than MSSA, except for rifampicin and chloramphenicol. However, when comparing CA-MSSA and CA-MRSA, we only found statistically significant differences when comparing resistance to ciprofloxacin, erythromycin, gentamicin, and cotrimoxazole.

## 4. Discussion

Although previous Indian studies have reported CA-MRSA prevalence of 4.6–10.6% [17–19], a study performed in the urban area of Bangalore, not far away from the site of our study, reported that 22.5% of 1000 healthy individuals were carriers of SA, and in 72.7% of them, SA was methicillin resistant [20]. The results of the Bangalore study and the high proportion of CA-MRSA found in our rural setting suggest that MRSA is replacing MSSA as the principal cause of CA-SA infections in India, but new studies in other parts of India are needed to confirm these findings. Similarly to the Bangalore study, we found high prevalence of CA-MRSA among children <5 years.

In a European study, the odds ratio for mortality of MRSA blood stream infections was 80% higher than MSSA blood stream infections, and the total costs attributable to excess hospital stays for MRSA were 44 million euros [21, 22]. With more than 1.2 billion people, 55% of them living in poverty, and a public health system in crisis [23], the effect of the MRSA spread into the Indian population in terms of deaths and costs can be desolating. Moreover, in this era of globalization, the spread of MRSA can affect other countries with lower levels of MRSA in the community. In two recent studies in Ireland and Japan, isolation of MRSA was observed in patients who had recently travelled to India [24, 25].

As described previously [2], methicillin resistance was associated with resistance to other antibiotics. In the present study, CA-MRSA had higher levels of resistance to ciprofloxacin, erythromycin, gentamicin, and cotrimoxazole than CA-MSSA. The high proportion of CA-MRSA must be considered when initiating empirical treatment for SSTI and other SA-related infections. In many resource-limited settings, it is not possible to obtain identification and antibiotic susceptibility of bacterial infections because of the scarcity of microbiology laboratories, so health-care professionals must decide antibiotic regimens according to epidemiological data and their clinical judgment. According to the results of this study, beta-lactam antibiotics alone may not be useful if we want to cover SA. In addition, antibiotic susceptibilities of CA-MRSA vary considerably in different parts of the world [10]. Empirical treatment of SSTI and other SA-related infections should be based on the prevalence of CA-MRSA and antibiotic susceptibilities of MRSA in the region. In another Indian study, the levels of CA-MRSA resistant to vancomycin, linezolid, clindamycin, and gentamicin were low, and ciprofloxacin and cotrimoxazole resistance was common [26]. These results are in accordance with our findings, suggesting that this information can be used to select empirical treatment in India. We also found that most of CA-MRSA and HA-MRSA strains were susceptible to doxycycline and chloramphenicol, so these antibiotics could be used when there are risk factors for HA-MRSA. 

The spread of MRSA in the community can have important implication for hospital infection control programmes. When the prevalence of MRSA increases in the community, CA-MRSA strains tend to replace HA-MRSA in health-care settings [27], making infection control measures less effective for reducing the prevalence of MRSA.

The study has some limitations. We did not perform typing of the MRSA strains in order to discriminate specific strains of CA and HA-MRSA [28]. Previous studies have found that the majority of CA-MRSA strains in India carry the Panton-Valentine leukocidin (PVL) virulence factor and has staphylococcal cassette chromosome mec (SCCmec) type IV and SCCmec type V [29, 30]. In a study performed in Mumbai, SCCmec type IV strains were typically resistant to gentamicin, and SSCmec type V strains were typically gentamicin susceptible [29]. In our study, most of CA and HA-MRSA were gentamicin susceptible so it is possible that SCCmec type V strains were predominant in our site, but new studies using genotypic typing are needed to investigate the predominant clones of CA-MRSA in India.

## 5. Conclusions

This study indicates that MRSA is replacing MSSA in CA-SA infections in this area of India. The high prevalence of MRSA in the community has important implication for the clinical management of SSTI and other SA-related infections and for hospital infection control programmes. If these findings are confirmed by other studies, the spread of MRSA in the community can have devastating public health consequences in terms of mortality and costs in India.

## Figures and Tables

**Table 1:** Percentage susceptibilities to antimicrobial agents of *Staphylococcus aureus. *

	Ciprofloxacin	Erythromycin	Clindamycin	Doxycycline	Gentamicin	Rifampicin	Cotrimoxazole	Chloramphenicol
CA-SA, % (95% CI)	25.2 (18.2–33.8)	58.6 (49.4–67.3)	87.1 (79.6–92.1)	93.7 (87.3–97)	74.6 (65.9–81.7)	91.4 (82.8–95.9)	55.2 (46–64)	95.6 (89.8–98.2)
HA-SA, % (95% CI)	19.8 (12.4–29.9)	62 (50.8–72.1)	75.9 (65.2–84.2)	83.6 (72.6–90.7)	78.2 (67.6–86.1)	91.1 (80.1–96.3)	58.8 (47.6–69.1)	90.9 (82–95.6)
CA-SA versus HA-SA, *P* value	0.368	0.634	0.045	0.03	0.56	0.953	0.619	0.189
CA-MSSA, % (95% CI)	42.9 (28.8–58.1)	70.7 (55.1–82.6)	92.3 (78.5–97.5)	97.5 (84–99.7)	92.9 (79.9–97.7)	89.7 (72.2–96.7)	78 (62.8–88.2)	92.7 (79.5–97.6)
HA-MSSA, % (95% CI)	33.3 (17.5–54.1)	82.6 (61.6–93.4)	95.7 (74.4–99.4)	95 (71.4–99.3)	95.7 (74.5–99.4)	100 (93.5–81.5)	69.6 (48.3–84.8)	95.2 (72.5–99.3)
CA-MSSA versus HA-MSSA, *P* value	0.446	0.292	0.605	0.611	0.654	0.17	0.452	0.698
CA-MRSA, % (95% CI)	15.6 (9–25.6)	52 (40.7–63.1)	84.4 (74.4–91)	91.5 (82.3–96.2)	64.5 (53–74.5)	92.3 (81.1–97.1)	42.7 (31.9–54.1)	97.3 (89.6–99.3)
HA-MRSA, % (95% CI)	14 (7.1–25.8)	53.6 (40.5–66.2)	67.9 (54.5–78.8)	78.7 (64.6–88.2)	70.9 (57.5–81.4)	87.2 (72.5–94.6)	54.4 (41.3–66.9)	89.3 (78–95.1)
CA-MRSA versus HA-MRSA, *P* value	0.803	0.859	0.024	0.046	0.439	0.417	0.182	0.063
MSSA, % (95% CI)	39.4 (28.3–51.7)	75 (62.9–84.1)	93.5 (83.9–97.6)	96.7 (87.5–99.2)	93.8 (84.6–97.7)	93.5 (81.4–97.9)	75 (62.9–84.1)	93.5 (83.9–97.6)
MRSA, % (95% CI)	14.9 (9.8–22.1)	52.7 (44.1–61.1)	77.4 (69.5–83.8)	86.4 (78.9–91.6)	67.2 (58.6–74.7)	90.1 (81.9–94.8)	47.7 (39.3–56.3)	93.8 (88–96.9)
MSSA versus MRSA, *P* value	<0.001	0.003	0.006	0.032	<0.001	0.51	<0.001	0.947
CA-MSSA versus CA-MRSA, *P* value	0.001	0.05	0.231	0.216	0.001	0.684	<0.001	0.252
HA-MSSA versus HA-MRSA, *P* value	0.046	0.016	0.009	0.1	0.016	0.122	0.212	0.418

CA: community associated; HA: health-care associated; SA: *Staphylococcus aureus*; MSSA: methicillin-susceptible *Staphylococcus aureus*; MRSA: methicillin-resistant *Staphylococcus aureus*; CI: confidence interval.
